# Assessing the performance of artificial intelligence in detecting electrographic status epilepticus when human experts often disagree

**DOI:** 10.3389/fneur.2026.1833203

**Published:** 2026-08-11

**Authors:** Khalid Alsherbini, Michelle Armenta Salas, Suganya Karunakaran, Archit Gupta, Baharan Kamousi, Raymond Woo, Veeresh Kumar N. Shivamurthy, David King-Stephens, Lawrence J. Hirsch, John Stern, Ioannis Karakis, Vikram R. Rao, Farid G. Sadaka, Parshaw J. Dorriz, Josef Parvizi

**Affiliations:** 1 Department of Neurology, Banner Health, University of Arizona, Phoenix, AZ, United States; 2 Ceribell Inc., Sunnyvale, CA, United States; 3 Department of Neurology, Trinity Health, University of Connecticut, Hartford, CT, United States; 4 Department of Neurology, University of California—Irvine, Irvine, CA, United States; 5 Department of Neurology, Yale School of Medicine, New Haven, CT, United States; 6 Department of Neurology, University of California—Los Angeles, Los Angeles, CA, United States; 7 Department of Neurology, Emory University, Atlanta, GA, United States; 8 Department of Neurology and Weill Institute for Neurosciences, University of California, San Francisco, San Francisco, CA, United States; 9 Department of Adult Critical Care, Mercy Hospital St. Louis, St. Louis, MO, United States; 10 Department of Neurology, Providence Health, Mission Viejo Medical Center, Mission Viejo, CA, United States; 11 Department of Neurology, Keck School of Medicine, University of Southern California, Los Angeles, CA, United States; 12 Department of Neurology and Neurological Sciences, Stanford University, Palo Alto, CA, United States

**Keywords:** artificial intelligence, EEG, electrographic status epilepticus, interrater agreement, reference standard

## Abstract

**Objective:**

Generating reference standards to train and evaluate the accuracy of artificial intelligence (AI) algorithms poses a significant challenge. Particularly when interpreting complex signals like electroencephalography (EEG), where interrater variability is considerable. We aimed to characterize the impact of interrater variability when evaluating the performance of an AI algorithm for detecting electrographic status epilepticus in point-of-care (POC) limited-montage EEG.

**Methods:**

We analyzed 604 EEGs collected using a POC EEG system (Ceribell Inc.). Each EEG was independently reviewed by 5–7 blinded experts, who annotated seizures and completed standardized assessments. The EEGs were later analyzed by the Clarity AI algorithm (version 7), trained on separate EEG dataset. Interrater agreement was assessed using Gwet’s AC1. Sensitivity and specificity for detecting electrographic status epilepticus (ESE) were estimated for the reviewers and the AI algorithm. Multiple evaluation schemes were employed, including simple majority consensus of the full group (group majority), simple majority using leave-one-out analysis, and 2-of-3 majority with bootstrap sampling.

**Results:**

Of 604 POC EEGs, the group majority identified 8 cases (1.3%) meeting ACNS criteria for ESE and 14 (2.3%) with seizures, the rest were classified as normal/slowing (80.1%), highly epileptiform patterns (6.6%), or other findings (4.3%). Twentynine cases lacked consensus interpretation. Interrater agreement among reviewers was 0.67–0.68. Compared to the group majority, the AI algorithm showed higher sensitivity (median 100%) with lower specificity (93.5%) than individual reviewers (median sensitivity 60%, specificity 98.7%). Using all possible 3-reviewer combinations to define majority agreement, the number of EEGs identified as ESE varied widely (4–18 cases, median = 10). The AI algorithm consistently achieved significantly higher sensitivity than external human reviewers (71.4% vs. 50%, *p* < 0.001). However, the AI’s specificity, while still high (median = 93.9%), was slightly lower than that of human reviewers (median = 98.4%, *p* < 0.001), though the AI’s specificity had consistently narrower spread.

**Discussion:**

This study highlights the challenges of defining the correct answer for EEG interpretation, especially for ESE, and its consequences for evaluating seizure detection AI tools. Future work should explore how AI assistance impacts human interpretation, particularly in reducing interrater variability across a broader range of EEG patterns.

## 1 Introduction

The validity of electroencephalography (EEG) as a diagnostic tool depends on reliable interpretation. Due to EEG’s complex signals and subtle features, the standard practice of visual inspection can be challenging. The initial development of the Critical Care EEG Monitoring Research Consortium (CCEMRC) aimed to provide standardization across the clinical practice of EEG monitoring (1, 2). This and other groups have raised the important problem of high interrater variability in EEG interpretation that ranges from fair to substantial when evaluating EEGs for seizures and epileptiform discharges (2–4). These investigations have identified a wide range of interpretation agreement (5–8), with more recent studies in large datasets finding fair (kappa = 0.34) pairwise chance-corrected agreements for seizures (4). It is known that interrater reliability varies significantly depending on what patterns or features are being labelled (6–9), the experience and training level of the reviewers (1–3), and whether the reviewers are knowledgeable about the EEG terminology guidelines (3–5).

Given the concerning problem of high interrater variability in EEG interpretation, the “gold standard” of EEG interpretation has traditionally relied on a consensus diagnosis reached by at least two of three experts; the ultimate “ground truth” would require access to all available information including clinical history, including past and future EEG findings. However, how this majority consensus can vary depending on the specific combination of 3 experts remains unknown. While standardized EEG terminology and criteria with full-array recordings have resulted in higher interrater agreement, no systematic study has examined how majority agreement may vary depending on which specific EEG reviewers are chosen to interpret these EEGs. Moreover, while previous studies have explored some of the underlying factors that impact interrater variability (9), it has remained to be determined how the problem of interrater variability impacts the training and evaluation of artificial intelligence (AI) algorithms intended to provide automatic interpretation of the EEGs.

In this study, we address these unknowns by focusing specifically on electrographic status epilepticus (ESE), since the accurate diagnosis of ESE (by EEG experts and the AI algorithms) has important, urgent clinical implications and will lead to immediate treatment decisions; over-calling the diagnosis could lead to potentially harmful escalation of treatment and under-calling the diagnosis may result in development of refractoriness, prolonged intensive care unit and hospital stays, and avoidable brain injury. Most importantly, the definition of ESE has gone through several iterations (10, 11) and although recent guidelines have made efforts to unify them (12, 13), its timely diagnosis and management largely depends on obtaining timely EEG (14–16).

The “ground truth” for diagnosing ESE relies on majority agreement among epileptologists reviewing full-montage recordings in the context of clinical and ancillary diagnostic data, often including response to interventions. However, the emergence of point-of-care (POC) EEG systems for the rapid diagnosis of ESE with fewer electrodes and integrated AI interpretation raises an hitherto unresolved question: what is the extent of interrater variability among expert epileptologists in diagnosing ESE in POCEEGs, and how should the accuracy of ESE detection by AI algorithms be evaluated in light of that variability?

In this investigation, we address this question with a systematic approach using a dataset of 604 POCEEGs interpreted by a group of expert reviewers. We explore interrater variability across different groups of experts and how AI and expert reviewers differ depending on the constituents of readers forming the majority opinion. The two aims of the study were (i) to characterize the practical implications of interrater variability for determining presence of ESE in the limited montage POCEEG recordings and (ii) to explore the impact of inter-rater variability on the evaluation of AI interpretation of these EEGs.

We are mindful that ancillary clinical information about each patient, including follow-up, would have been valuable in establishing a “ground truth” diagnosis. However, we did not have access to follow-up data because the EEGs in our dataset were encrypted and de-identified, and more importantly, the aim of our study was not to address the performance of AI algorithm compared to “ground truth” but to “majority agreement” of human readers in the context of interrater variability among these human experts. A comparison between AI and humans would be inherently biased if the algorithm was asked to analyze EEG data alone but human experts were given access to clinical context. Accordingly, we designed the study to compare the sensitivity and specificity of the AI to human readers who were likewise limited to EEG data only, thereby avoiding the introduction of bias by providing clinical information to humans but not to the algorithm.

## 2 Materials and methods

### 2.1 Dataset selection and EEG reviews

We compiled a retrospective dataset of sequentially sampled clinical POCEEG recordings with the Ceribell EEG system (Sunnyvale, CA), which includes a battery-powered pocket-sized recorder and a disposable headband with 10 scalp electrodes covering the circumference of the head laterally producing 8 channel bipolar montage. The EEG is displayed on the recorder as well as on a cloud portal with a fixed bipolar montage.

Each hospital system contributed EEG studies conducted in the course of 2–4-months. Patients were 18 years and older with a recording duration of at least 15 min, recorded in the emergency department (ED), intensive care unit (ICU) or non-ICU hospital wards. A total of 604 cases were included across three hospital systems: Mercy Health (2 tertiary care hubs, 2 community spokes), Providence Mission Viejo (1 tertiary care hub, 1 community spoke), and Yale New Haven Health System (1 tertiary care hub, 4 community spokes).

Central IRB approval was obtained for the review of anonymized clinical EEG recordings from Ceribell’s database (WCG, Num.: 1-1,766,932-1). Seven reviewers were recruited to participate, all with over a year post-fellowship experience (clinical neurophysiology or epileptology) and were experienced in reading Ceribell EEGs in their clinical practices (Supplementary Table 1). While reviewers were not required to complete self-assessment test administered through CCEMRC, all reviewers, were trained in American Clinical Neurophysiology Society’s (ACNS) standardized terminology, and before reviewing the EEGs, familiarized themselves with the new ACNS definition of ESE on the screen prior to rating each EEG. Reviewers had access to the anonymized EEG but no clinical information about the cases. Reviewers were asked to annotate the start and stop of all seizures on the EEG through an online portal. They also completed a standardized questionnaire for each case noting background information, presence of common EEG patterns and seizures, as well as categorization of whether ESE was present according to ACNS criteria (12).

The review process was split into two phases and the sequence of EEGs in each set of cases was randomized for each reviewer. In the first phase, five reviewers read and annotated 222 EEGs from the Mercy hospital system. In the second phase, the same five reviewers plus an additional two reviewers read and annotated 382 EEGs from the other two hospital systems, randomized within each system (Providence Mission Viejo = 221, Yale New Haven Health System = 161).

### 2.2 Data analysis

All 604 EEGs were analyzed with the same Clarity AI algorithm (v. 7). Clarity AI is a seizure-burden monitoring algorithm that uses machine learning models to detect seizures and estimates the seizure burden as the proportion of time with seizure activity from a rolling 5-min window, which updates every 10 s (17). It also includes an ESE monitor component that runs in parallel and generates a separate output, cleared by the FDA to diagnose ESE based on ACNS guidelines. For the Clarity AI 5-min seizure burden (SzB) output, we obtained the maximum SzB per recording. For the ESE monitor, we obtained a binary output per EEG, where a positive value indicates 10 min of continuous seizure activity at any point or ≥20% of any one-hour window with seizures. The algorithm was independently developed by Ceribell, current and previous versions of the algorithm were trained and validated on independent data using their own 8-channel headband.

Reviewers’ responses were tabulated and pooled together across both phases and all EEGs. We categorized the responses to the presence of ESE if the reviewer chose that the seizure activity “meets ACNS criteria.” For the AI algorithm label, we determined the algorithm endorsed ESE if either (1) the maximum Clarity SzB was ≥90%, or (2) the ESE Monitor output was positive.

### 2.3 Interrater agreement in POC-EEG reading

To assess agreement among reviewers, we estimated the percentage agreement (PA) and Gwet’s AC1/2 coefficients using the file-level determinations. Gwet’s AC1/2 coefficient is robust to underestimations of agreement in low-prevalence diseases (18), like ESE, and was recently reported in other EEG agreement assessments (19, 20). We evaluated these agreement metrics among the 5 reviewers who evaluated all 604 EEGs and among the 7 reviewers in the subset of 382 EEGs.

### 2.4 Performance for detecting ESE

We simulated how an AI algorithm may be assessed in comparison to different gold-standard (“final answer”) schemes: single reviewers and majority consensus from the large pool of reviewers (including all reviewers and excluding the index reviewer), and majority consensus of 3 reviewers (using all different possible combinations of 3 reviewers). For each scheme, we estimated the sensitivity and specificity for detecting ESE. Non-parametric tests were used to compare the distributions of sensitivities and specificities for reviewers and the AI algorithm.

To assess individual reviewers and the AI algorithm, we used the majority consensus (i.e., when proportion of reviewers that called ESE was greater than 50%) of all available reviewers to generate a *group majority agreement*. We evaluated the accuracy of each individual reviewer as well as Clarity against this majority agreement. However, since the individual reviewers would be part of the majority-building group, we performed a sub-analysis where each individual reviewer’s response was excluded. The AI algorithm and the individual reviewers were evaluated against the *leave-one-out* majority *agreement* where neither was part of majority-building group.

Next, to systematically compare how interrater variability impacts detection of ESE using a majority consensus standard, we formed three-reviewer groups out of the pool of reviewers and applied a *2-of-3 majority agreement* to all possible 3-reviewer combinations. We used the 604 cases with 5 reviewers to generate 3-reviewer groups, using bootstrap resampling (*N* = 5,000), and evaluated the remaining two external reviewers as well as the AI algorithm against the 2-of 3 majority agreement.

Finally, as interrater variability is expected to not be homogeneous across all EEGs, but rather it may be primarily driven by a subset of EEGs that create higher disagreement among the human readers. To explore the performance of the algorithm when there was low variability between reviewers, we tabulated the percentage of reviewers that agreed that the EEG met criteria for ESE and selected the subset of cases where 80% or more of reviewers agreed an EEG was ESE or not ESE. We then estimated the overall performance of the AI algorithm among these cases. To adjust for the multiple tests performs, we adjusted for multiple-hypothesis tested in each statistic (sensitivity and specificity), using the Bonferroni–Holm correction.

## 3 Results

In 604 EEGs, 8 EEGs (1.3%) met ACNS ESE criteria according to a majority of reviewers, 14 (2.3%) had seizures, 40 (6.6%) had highly epileptiform patterns (HEPs)—i.e., generalized or lateralized periodic discharges, and lateralized rhythmic delta activity, 26 (4.3%) had other findings, the most common being burst suppression, and 487 (80.6%) had no epileptiform abnormalities. In 29 cases, no label had a clear majority, and these were used as “non-ESE” for performance evaluations. The median recording duration was 3.1 h [IQR = 1.96, 5.27]. Supplementary Table 2 shows general recording information of the cases, extracted from the anonymized database.

The percentage agreement (PA) on the file labels among the five reviewers that reviewed all EEGs files with a majority determination (*N* = 572) was 70.5%, while the Gwet’s AC1 agreement was 0.67 (0.65–0.70). In the dataset evaluated by seven reviewers with a majority determination (*N* = 359), the PA was 71.3% and Gwet’s AC1 was 0.68 (0.65–0.71).

### 3.1 Single reviewers and AI algorithm performance

Table 1 shows the summary of the individual reviewers’ ESE labels and their performance to detect ESE as determined by a *majority agreement* of the full group of reviewers when including and excluding the evaluated reviewer. Similarly, the AI algorithm output is shown for the majority agreement with and without the evaluated reviewer. When all reviewers were included, the sensitivities of the individual reviewers ranged from 12.5 to 100%, while the AI algorithm sensitivity was 87.5%, which was in the 80th percentile of the sensitivities of the human readers. The specificities of the reviewers ranged from 86.1 to 100%, and the algorithm’s specificity was 93.9%, which was in the 25th percentile of the human readers’ specificities.

**Table 1:** AI algorithm and individual reviewers’ performance for ESE detection.

Evaluated reviewer	Number of ESE cases	Sensitivity, %	Specificity, %
Majority agreement (including all reviewers)			
AI Algorithm	8	87.5	93.9
Rev. 1		75.0	99.0
Rev. 2		12.5	100
Rev. 3^a^		40.0	98.9
Rev. 4		100	86.1
Rev. 5		75.0	99.3
Rev. 6		87.5	98.3
Rev. 7^a^		80.0	99.5
Majority agreement (excluding index reviewer)			
Rev. 1	5	60.0	98.5
AI algorithm		100	93.6
Rev. 2	8	12.5	100
AI algorithm		87.5	94.0
Rev. 3^a^	4	25.0	98.7
AI algorithm		75.0	92.3
Rev. 4	3	100	85.4
AI algorithm		100	93.3
Rev. 5	5	60.0	98.8
AI algorithm		100	93.6
Rev. 6	4	75.0	97.7
AI algorithm		100	93.5
Rev. 7^a^	3	66.7	98.9
AI algorithm		66.7	92.1

ESE, electrographic status epilepticus; AI, artificial intelligence; Rev., reviewer.

a Reviewer only read 382 cases.

The top-half section, majority agreement (including all reviewers) shows number of ESE per the majority agreement, sensitivity and specificity metrics for clarity and each reviewer when including all available reviewers to determine the majority agreement. The bottom-half section, majority agreement (excluding index reviewer), shows the number of ESE cases and the performance metrics when excluding each of the reviewers from the majority agreement determination.

**Figure 1:**
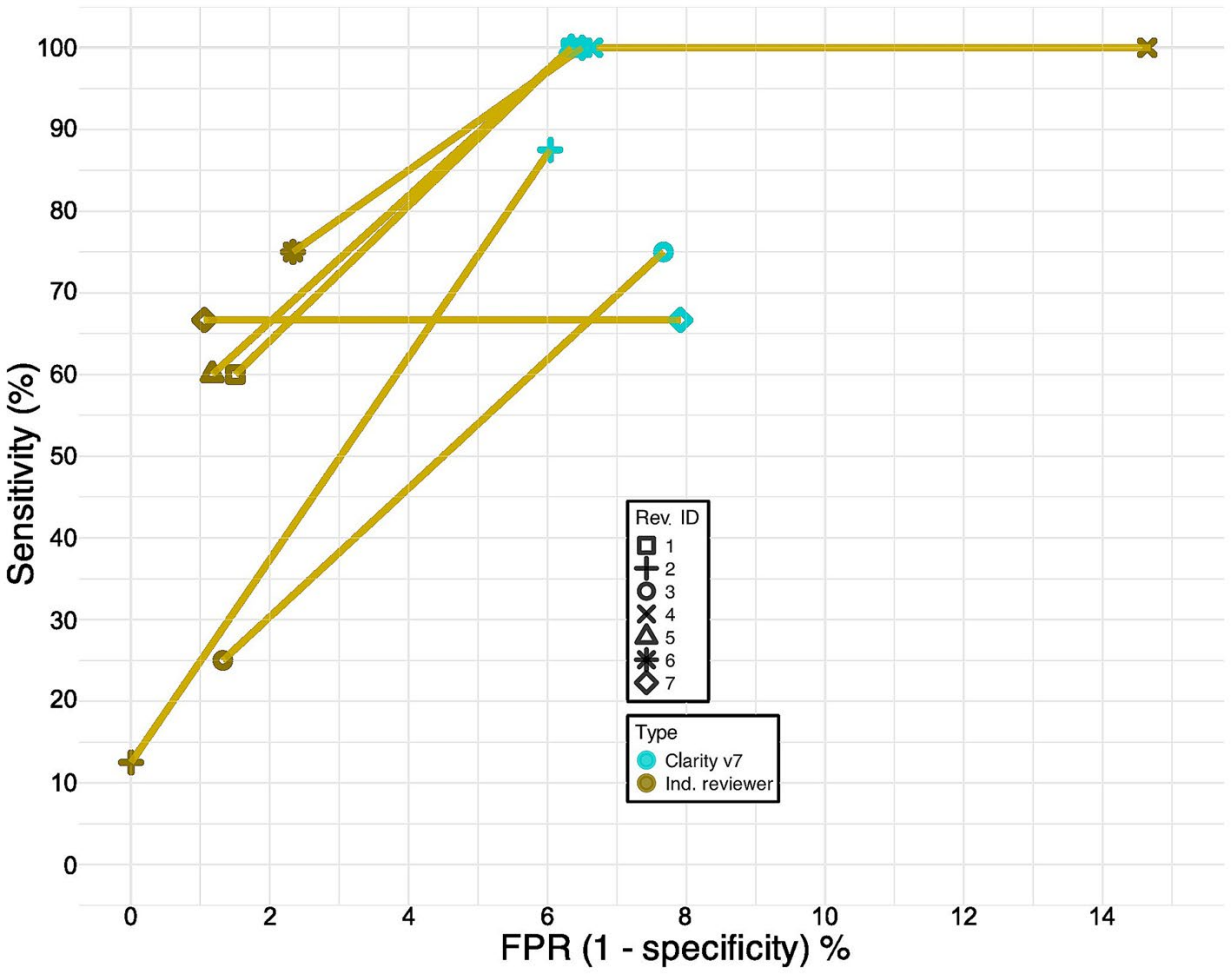
Distribution of performance from leave-one-out analysis. Reviewer’s and Clarity AI algorithm compared against the majority agreement excluding the index/left-out reviewer. The shape of the markers indicates the reviewer excluded from the majority, and the lines connect the corresponding pair of index/left-out reviewer and the Clarity AI algorithm, both evaluated against the same majority agreement.

When excluding the index reviewer, meaning the reviewer whose performance was being evaluated, to determine the *leave-one-out majority agreement*, the sensitivity of the individual reviewers had a median of 60% (IQR = 42.5–70.8%), and was significantly lower than the sensitivity of the AI algorithm [median = 100% (81.2–100), *p* = 0.039]. The specificities across reviewers were significantly higher than for the AI algorithm [median 98.7% (98.1–98.9) vs. 93.5% (92.8–93.6), *p* = 0.031]. Figure 1 illustrates the human readers’ and the AI algorithm’s performance when excluding the index reviewer.

### 3.2 External reviewers and AI algorithm compared to majority consensus of 3 reviewers

**Figure 2:**
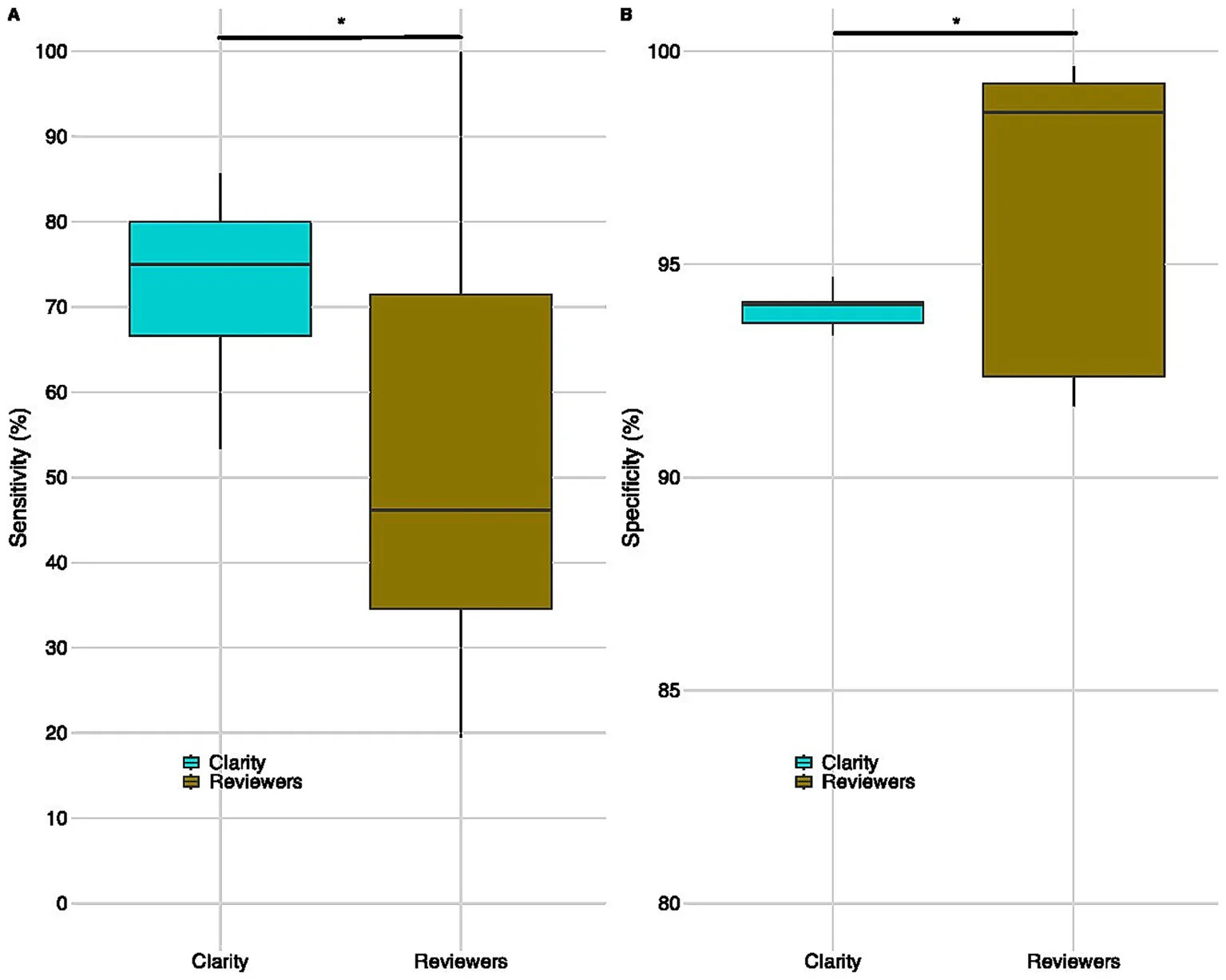
Performance for ESE detection for the AI algorithm (Clarity) and the external reviewers, using standard two-thirds consensus. **(A)** Sensitivity boxplot, with main body showing the 25th and 75th interquartile range and whiskers illustrating the range of values. **(B)** Specificity box plot. Each panel shows the performance for the AI algorithm (cyan) and the average among the external reviewers (brown). Top horizontal bar and asterisk show statistical significant difference between the distributions, after multiple comparison adjustment.

When iterating over all possible groups of 3 reviewers, the number of ESE cases ranged from 4 to 18 [median = 10, (7–13)], depending on the group of reviewers that generated the *2-of-3 majority*. In the bootstrapping analysis, the AI algorithm had significantly higher sensitivity than the average external reviewers, meaning those who were not part of the 3-reviewer group being tested [median sensitivity = 71.4% (66.7–80) vs. 50% (34.6–71.4), *p* < 0.001]. In contrast, the AI algorithm had significantly lower specificity than the external reviewers, although both reviewers and the algorithm had very high specificities and the spread of the distribution was much narrower for the AI algorithm [median Spec. = 93.9% (93.6–94.1) vs. 98.4% (92.4–99.2), *p* < 0.001]. Figure 2 summarizes the distribution of estimated sensitivities and specificities for the AI algorithm and the reviewers.

### 3.3 AI algorithm compared to group majority consensus in high-agreement cases

Supplementary Figure 1 shows the distribution of all 604 cases grouped by the proportion of reviewers that agreed whether the case met ACNS ESE criteria. Overall, we found that in 96.4% of cases (582/604), the large majority of reviewers (80–100%) agreed whether or not a case was ESE. Using the subset of 582 cases with high agreement, we examined the performance of the AI algorithm. Among these cases, the reviewers agreed that 2 cases met ESE criteria. The algorithm captured both cases, for a 100% sensitivity, and had 28 false positives, for a 95.2% specificity.

### 3.4 Impact of reviewers’ experience and EEG patterns

Next, in the subset of 382 cases with seven reviewers we varied the number of possible reviewers from 4 to 6, using bootstrap re-sampling (*N* = 5,000) to estimate the performance of external reviewers and the AI algorithm for detecting the ESE cases in each iteration.

**Figure 3:**
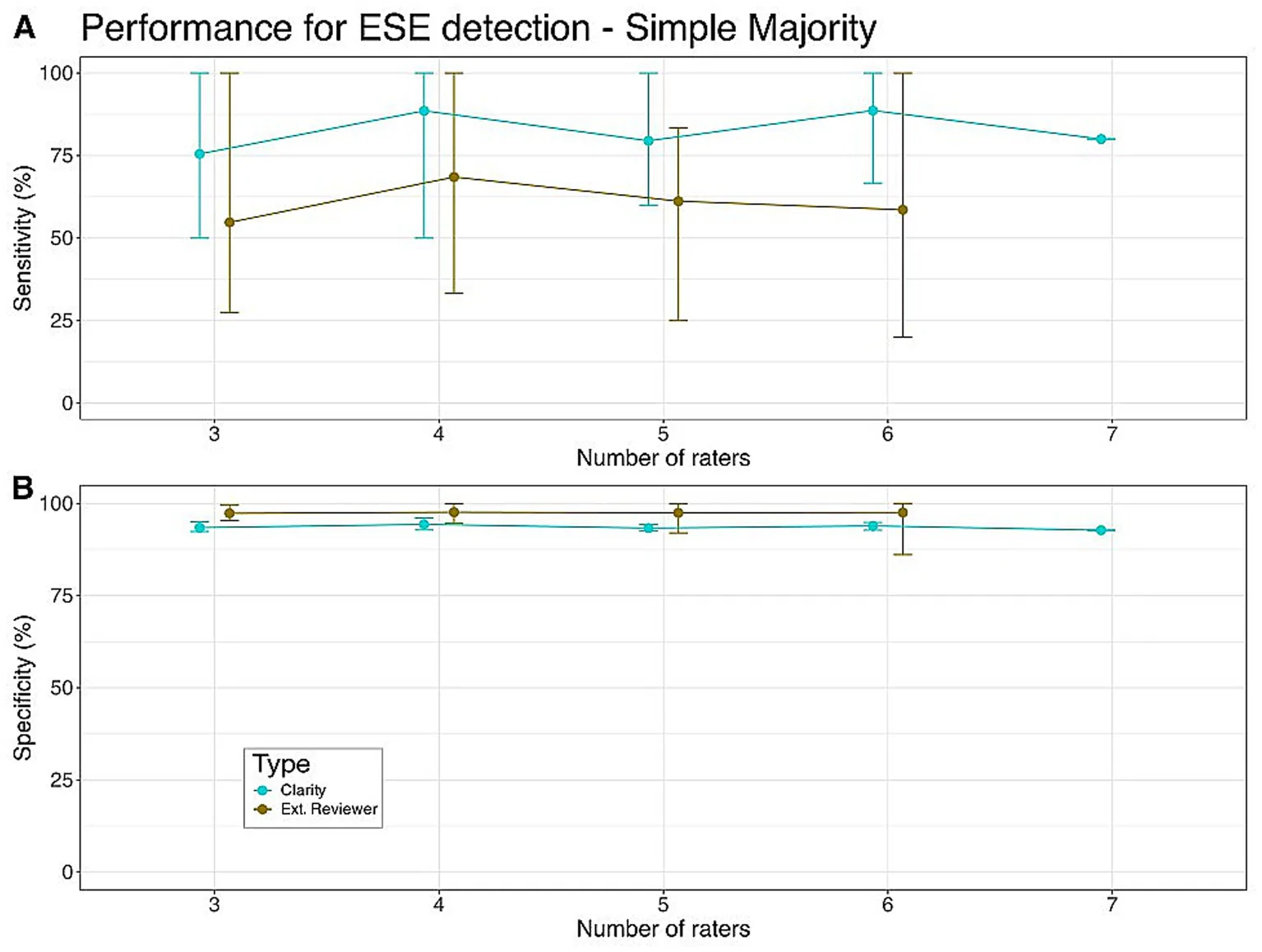
Estimation of performance for ESE detection as number of reviewers/raters varies. The performance was estimated from the resampled groups, where each group had the number of raters specified in the horizontal axis. The external reviewers were those outside the raters’ group. **(A,B)** Sensitivity and specificity, respectively. Color scheme as in Figure 2. All pairwise comparisons with data for both Clarity and the external reviewers were significant after multiple comparisons correction (*p* < 0.01).

Figure 3 summarizes sensitivity and specificity results for the AI algorithm and the reviewer against the *leave-one-out majority agreement*. Across all reviewer groups, the sensitivity for the AI algorithm was significantly higher than for the external reviewers (Supplementary Table 2, *p* < 0.001), and the variance in the distribution was also lower. For specificity, the performance was significantly lower for the AI algorithm than for the reviewers (Supplementary Table 2), although the variance was significantly lower for the algorithm. As the number of reviewers varied, we observed slight variations in the performance metrics, although none very significant. In cases where the majority requires a higher agreement (e.g., 75% when having four reviewers) the performance of both the AI algorithm and the external reviewers was higher. Further, when exploring which patterns were more likely to elicit ESE alerts by the algorithm, we found that cases identified by a majority of reviewers as ESE, seizure or HEP were far more likely to be alerted as suspected ESE, as shown in Supplementary Table 3.

There was notable variability in the patterns reported for cases not classified as ESE by human experts. Some reviewers identified them as containing highly epileptiform patterns, whereas others labeled seizures but noted that their duration did not meet ACNS criteria for ESE. For the cases without agreement on the non-ESE pattern we evaluated the patterns present, Supplementary Figure 3 shows a breakdown of these patterns, we found that all patterns were present across these cases, with the large majority having HEP, other abnormal or slowing patterns. It is important to note that each EEG case was followed by a question asking expert reviewers whether they would recommend treatment with antiseizure medications, including benzodiazepines. However, many reviewers expressed hesitation in making this determination without additional clinical context, such as patient history or prior medication administration.

Lastly, Supplementary Figure 4 shows the summary of a generalized mixed effects models, which accounts for fixed EEG and reviewer specific characteristics like recording duration, recording location and reviewers’ experience, as well as random effects due to individual files and reviewers. We explored whether any of these variables impacted whether an POCEEG was more likely to be labeled as ESE, outside of the inherent reviewer variability. Overall, we found that although certain POCEEGs were more likely to be labeled as ESE, the majority of the files did not inherently impact the label the file would receive. Although we recognize that more granular information on the years of experience and years of familiarity with the device, which we did not capture, might have resulted in a more measurable impact on interrater variability that was not captured with our current variables.

## 4 Discussion

In an era of rapid advancements in AI algorithms for medicine, our study addresses a critical and practical challenge: how to evaluate the accuracy of an AI algorithm (in our case, for detecting ESE) when significant interrater variability exists among the expert interpretations that define the correct final answer.

In our study, we show that the majority agreement of experts was highly dependent on the set of reviewers selected to establish consensus. Although overall reviewers’ agreement was moderate, the performance of reviewers, compared to majority agreement, varied significantly (12.5–100% sensitivity), moreso than the AI algorithm (66.7–100% sensitivity).

Our finding of significant inter-rater variability is in keeping with the existing literature documenting inter-rater variability when experts review data acquired with conventional EEG systems with full electrode sets, especially concerning the EEG waveforms that are in the gray zone between ictal and non-ictal states (i.e., the ictal—interictal continuum)—a pattern seen frequently in ICU patients with altered mental status. Interrater agreement varies across different EEG patterns (4, 8) and even after careful training, different experts may have different internal thresholds that will impact their reviews (21). The CCEMRC (22, 23) has acknowledged this issue and addressed it by developing training modules and certification programs. Standardized EEG terminology and criteria with full-array recordings have resulted in higher interrater agreement.

The substantial interrater variability in both individual reviewer performance and the number of EEGs labeled as ESE by majority consensus by the full group or through the leave-one-out is illustrated in Table 1, Figure 1. Some reviewers identified only unequivocal cases of ESE, resulting in high specificity but low sensitivity, whereas others labeled ESE more liberally, achieving higher sensitivity at the expense of specificity. When individual reviewers were evaluated against a robust majority standard (consensus of the full group), their overall performance declined, most notably due to reduced sensitivity. As such, experts evaluating a given AI algorithm, even when they have high confidence in their own interpretation (24), ought to recognize that their disagreement with the AI output may reflect their own divergence from the majority consensus rather than an error in the algorithm per se.

Variability in the sensitivity of individual reviewers may be akin to AI algorithm designed with different sensitivity thresholds. However, in real clinical practice, each EEG reader internally sets their own threshold. In contrast, the sensitivity of AI algorithms can be changed and tailored to address the clinical workflow needs: if used in the emergency settings, a high sensitivity is desired but in other conditions a higher specificity may be desired with explicit understanding about its implications. As shown in Figures 2, 3, across different methods to generate a majority agreement, the AI algorithm used in the current study performed with higher sensitivity for detecting ESE at the cost of specificity. Although we note that of the 604 EEGs, the Clarity AI algorithm flagged 43 cases as ESE, representing 7.1% of all studies. Of these 43 cases, 7 were confirmed true positives. Of the remaining 36 algorithmically flagged cases, only 2 contained no epileptiform activity whatsoever by any reviewer. The remaining 34 (i.e., representing 94% of so-called “false positives”) carried clinically meaningful EEG findings: 3 had short seizures by majority consensus, 17 had highly epileptiform patterns, and 13 had significant slowing. This is a critical distinction. These 34 cases were not erroneous alarms in any clinically meaningful sense, but they represented abnormal EEGs that warranted neurological attention. Labeling them “false positives” based solely on the absence of ESE would mischaracterize their clinical significance. True alarm fatigue arises from alerts that are clinically irrelevant. In this dataset, only 2 of 604 EEGs accounting for 0.3% of all EEG recordings analyzed generated an alert with no supporting epileptiform or abnormal findings. Overall, we consider that this trade-off is likely acceptable for an automated tool that screens for a low prevalence, but high morbidity, condition like ESE.

In real-world clinical practice, EEGs are typically interpreted by a single reviewer, often including varying degrees of clinical information and evolving EEG patterns. Given the substantial variability in experts’ ability to diagnose ESE when using reduced montage, as examined in this study, a patient’s care and welfare will critically depend on how closely the reviewer’s interpretation aligns with the majority agreement. In this context where expert interpretations are subject to substantial variability, and the single reviewer’s assessment may vary significantly from the majority agreement, AI algorithms may offer several key advantages. First, compared to a clinician who is not well-trained in reading EEGs, a well-validated AI algorithm will yield consistent output that is close to majority agreement, and thus offering the potential to reduce diagnostic uncertainty and supporting a more standardized clinical decision-making. Second, a stable AI system minimizes the variability associated with multiple human reviewers by always providing a consistent reading to the same EEG pattern. This reliability is particularly valuable in large-scale studies, automated analysis pipelines, and routine diagnostics, where consistency is critical. Third, while high interrater variability among experts complicates the establishment of a reference standard, a stable AI algorithm can serve as a consistent reference point, facilitating the identification of outlier interpretations, which in turn will contribute to improved quality assurance. Indeed on an simulation in our dataset of how AI + human may perform, that is if the individual reviewers were to only assess for ESE in the cases alerted for suspected ESE by the AI algorithm, we found that sensitivity would remain the same but specificity would improve (Supplementary Figure 5). Although hypothetical, this analysis may speak to the potential clinical utility for triage and optimizing resources. However, we are mindful that consistency in performance may not be advantageous if the AI is systematically biased to overcall or undercall.

This study also highlights an important issue rarely addressed in the field: the challenge of evaluating seizure-detection algorithms when the reference standard is variable and influenced by noise (21). The results of the algorithm’s performance on EEGs with high agreement among reviewers illustrate that a highly sensitive AI algorithm will follow closely reviewers’ consensus. This type of tool could potentially be used as a model of readers’ consensus, especially since most of these algorithms are trained from labels generated by multiple readers (9, 17). This is further demonstrated in the subanalysis with different number of reviewers used to establish the majority agreement, where the algorithm’s performance remained stable (Figure 3). However, no tool is perfect, and we note that even among the high-agreement EEGs the algorithm had false positives, demonstrated by the 95% specificity. While the performance of AI algorithms will undoubtedly improve with more training, clinical teams using such algorithms would need to determine acceptable performance trade-offs of potential misses and overcalls. For example, workflows incorporating AI algorithms with clinical suspicion and specific seizure burden thresholds have recently been proposed (25).

We are mindful that our study is not without limitations. First, as noted, our study used EEGs recorded with point-of-care EEGs with reduced number of channels that are always displayed in bipolar montage, which may have introduced some variability to the assessments. For instance, it is possible that the lower experience of the reviewers reading reduced-montage EEGs compared to conventional EEGs may have impacted the agreement rates among reviewers. However, we believe the slightly lower agreement rates observed in our study cannot be attributed solely to the use of fewer electrodes or the inability of reviewers to manipulate the EEG display using different montages. Many of the EEGs analyzed were from patients presenting with neuroemergencies. These recordings often feature patterns with severe abnormalities, which are associated with lower agreement rates, even when captured with full montage. Due to the nature of the reduced montage, our results may not generalize to full-montage or even other forms of reduced montages. However, we note that the interrater agreement values reported in our study are on par with the ones reported in the literature for full conventional EEGs where specific definitions of status epilepticus (19, 24) were included.

A second limitation of our study is that neither the human reviewers nor the AI had access to clinical information. In real-world settings, EEG interpreters often rely on clinical context to guide their diagnostic decisions, which may have changed their accuracy. For instance, a compelling clinical history may increase suspicion for ESE, making interpreters more likely to diagnose it. This could enhance sensitivity but potentially reduce specificity. Conversely, certain clinical factors might lead interpreters to be more cautious and less likely to diagnose ESE, even when EEG findings are suggestive. In a recent survey study of electroencephalographers, Nascimiento et al. (26) suggested the possibility of bias in EEG interpretation if clinical information is reviewed before the reading of EEGs. The bias may take the form of perception, where some patterns may be more readily interpreted as pathological when clinical history is known, and of interpretation, where patterns that would be identified regardless of clinical history may be raised as more (or less) concerning due to certain aspect of the clinical history.

A third limitation of the study is that the number of positive ESE cases was very small when stringent agreement thresholds were applied: only one case met the >90% agreement criterion, and just four exceeded 60% agreement. This raises the possibility that our results may be disproportionately influenced by a small number of such cases. Future work using larger cohorts with datasets enriched for ESE will be necessary to address this limitation.

Additionally, the EEG reviewers contributing to this study were either established experts or well-trained early-career EEG specialists. In routine clinical practice, many readers may have substantially less training or experience. It would therefore be valuable to examine in future studies how “real-world” EEG readers might perform relative to the AI, as well as to consider how diagnostic decisions could be influenced when less experienced readers rely on or are guided by such AI systems, particularly in light of the model’s reduced specificity.

In closing, we like to emphasize that human and AI EEG readers should not be seen as competitors; rather, AI-based algorithms are designed to support and enhance human decision-making and not to replace it. Future research should prioritize integrating human and AI interpretations in a manner that reflects real-world clinical workflows, while also evaluating how AI affects interrater variability and expanding the scope beyond ESE to include other clinically relevant EEG patterns.

## Data Availability

The datasets presented in this article are not readily available because the EEG datasets are not publicly available, and we do not have permission to share the raw data under the terms of the IRB determination. The reviews generated for this study are the property of Ceribell, Inc., but reasonable requests may be considered by the corresponding author. Requests to access the datasets should be directed to Josef Parvizi, jparvizi@stanford.edu.
