# Supplementary material for: Assessing the performance of artificial intelligence in detecting electrographic status epilepticus when human experts often disagree
Source: Front Neurol. 2026 Aug 11;17:1833203. doi: 10.3389/fneur.2026.1833203 (PMC13504800; doi:10.3389/fneur.2026.1833203)
Supplement: Supplementary file 1 [file Data_Sheet_1.PDF]

Supplementary Material

1 Supplementary Data

1.1 EEG reviews categories

Reviewers were presented with a standardized questionnaire for each EEG in which the reviewers noted EEG background continuity, the presence of focal slowing, the presence of periodic patterns or lateralized rhythmic activity, the presence of seizures that did not contribute to a finding of electrographic status epilepticus (ESE), as well as a file-level categorization of the most severe electrographic finding throughout the entire recording. The questionnaire also asked reviewers to assess the EEG for the presence of ESE by choosing one of the following four categories: 1) ESE present based on seizure duration and American Clinical Neurophysiology Society (ACNS) criteria(1); 2) ESE present based on frequency of discrete seizures without returning to normal background and ACNS criteria; 3) ESE present based on the ictal or paroxysmal patterns that do not meet ACNS criteria for status epilepticus but are sufficient for recommendation to treatment for possible status epilepticus; and 4) ESE absent.

1.2 Recording duration and agreement

We examined any potential correlation between the recording duration and the agreement between reviewers on whether an EEG had ESE present or absent. In general, there wasn't a strong correlation between absolute EEG duration and proportion of agreement between reviewers (rho = -0.076, p = 0.06, Spearman correlation). Grouping the cases by high and low agreement, i.e., those where at least 80% of the available agree on a case being ESE/no-ESE vs those with lower than 80%. **Supplementary Figure 2** shows the scatter plot of EEG duration versus agreement

1.3 Assessing impact of EEG and Reviewers' characteristics

To explore whether characteristics of the EEG, like duration or recording location, and the reviewers, like experience, had any impact on a file being labeled as electrographic status epilepticus (ESE), as well as if certain outlier EEGs or reviewers impacted the labels. To avoid experience categories with only one reviewer, we categorized the reviewers' experience as 1-10 years and ≥15 years.

Using R Studio software (2025, Posit Software, PBC, Boston, MA), we fitted a generalized mixed effects models to assess the impact of EEG duration, recording location and reviewer's experience as fixed effects, and the EEGs and reviewers as random effects. **Supplementary Figure 4** summarizes the model, which explained substantially the variability in the data (conditional $R^2 = 0.977$), with the part explained by the fixed effects (marginal R^2) being 0.005. Within the model, the fixed effects were not statistically significant, as noted in the table, and the EEG file itself (coded as fileID) was the random effect that had the largest positive impact.

When examining the effect of individual cases, there were 100 files that their effect on the ESE label was large (greater than +/- 2), which means something in these files, independent of duration, recording location or reviewer type, impacted whether the file would be labeled as ESE. Upon inspection of the patterns, these cases were more likely to have labels of HEP, Seizure and ESE,

compared to those EEGs with smaller effect per the model. Aligned with this finding, was the fact that all of these files had positive effect, meaning their impact was for the EEG to have an ESE label. Note that these cases were part of the primary analyses and the linear mixed model analysis, but due to biased nature of this subset, we did not evaluate the performance of the reviewers or the algorithm in this subset of cases.

1.4 AI+human reviews

To evaluate the hypothesis that an AI tool like the algorithm analyzed in this study could potentially improve the reviewers' performance and agreement. We estimated the individual reviewer's performance with the leave-one-out analysis, if the index reviewer were to evaluate for presence of ESE on cases only alerted by the algorithm. We evaluated the changes in performance with a pairwise Wilcoxon test. The updated reviewers' performance is show in **Supplementary Figure 5**. Overall, we found that individual reviewers' sensitivity would have remain the same, but their specificity would have significantly improved ($p = 0.031$).

2 Supplementary Figures and Tables

2.1 Supplementary Tables

Supplementary Table 1. Demographics and experience summary of expert reviewers

	Rev 1	Rev 2	Rev 3	Rev 4	Rev 5	Rev 6	Rev 7
**Sex**	M	M	M	F	M	F	M
**Years post-fellowship***	1-5	>20	>20	15-20	5-10	1-5	1-5
**Fellowship training**	CNP & Epilepsy	Epilepsy/EEG	CNP	CNP	Epilepsy	Epilepsy	Epilepsy

*At the time when reviews finished, fall of 2024

CNP: clinical neurophysiology; EEG: electroencephalography

Supplementary Table 2. EEG recording characteristics

	Consecutively sampled cases
**Total**	604*
**Recording duration**	
**Median [25th, 75th] *hours***	3.1 [1.96, 5.27]
**Min-max *hours***	0.25 – 20.35
**Recording location†**	
**ED (%)**	165 (27.3%)
**ICU/Floor (%)**	437 (73.4%)
**Recording start time**	
**Weekday workhours (8a-5pm) (%)**	167 (27.6%)
**Weekend or weekday after-hours (>5pm & <8a) (%)**	437 (72.4%)
**Primary EEG indication***	
**Prior seizure, suspicion of seizure or history of epilepsy**	279 (49.2%)
**Cardiac arrest (post-ROSC)**	80 (14.1%)
**Toxic/Metabolic**	56 (9.9%)
**AMS, undifferentiated encephalopathy or altered consciousness**	47 (8.3%)
**ABI hemorrhagic or ischemic (e.g., stroke, ICH, SAH)**	47 (8.3%)

†2 cases didn't have location information

*As entered at bedside recorder during at start of EEG study

ED: emergency department; ICU: intensive care unit; ROSC: return of spontaneous circulation; AMS: altered mental status; ABI: acute brain injury; ICH: intracerebral hemorrhage; SAH: subarachnoid hemorrhage

Supplementary Table 3. Mean and standard deviation of sensitivity and specificity of external reviewers and the AI algorithm.

Number of reviewers in each column represents the number of experts considered to generate the consensus ground truth.

	3 reviewers	*4 reviewers*	*5 reviewers*	*6 reviewers*
External reviewers' sensitivity	*54.9% (19%)*	*68.0% (19%)*	*61.4% (16%)*	*59.2% (26%)*
AI algorithm's sensitivity	*75.6% (16%)*	*88.2% (16%)*	*79.7% (14%)*	*88.9% (13%)*
External reviewers' specificity	*97.3% (1.8%)*	*97.6% (2.2%)*	*97.3% (3.2%)*	*97.4% (4.7%)*
AI algorithm's specificity	*93.4% (0.7%)*	*94.3% (0.8%)*	*93.3% (0.5%)*	*93.9% (0.6%)*

Supplementary Table 5. Breakdown of patterns by majority consensus in cases alerted for ESE by the algorithm (N = 43) and without ESE alert (N = 561)

	**Total**	Status	Seizure	HEP	Abnormal	Normal/Slow	No consensus
ESE by AI	**43**	7 (16.3%)	3 (7%)	17 (39.5%)	0 (0%)	13 (30.2%)	3 (7%)
non-ESE by AI	**561**	1 (0.2%)	11 (2%)	23 (4.1%)	26 (4.6%)	474 (84.5%)	26 (4.6%)

2.2 Supplementary Figures

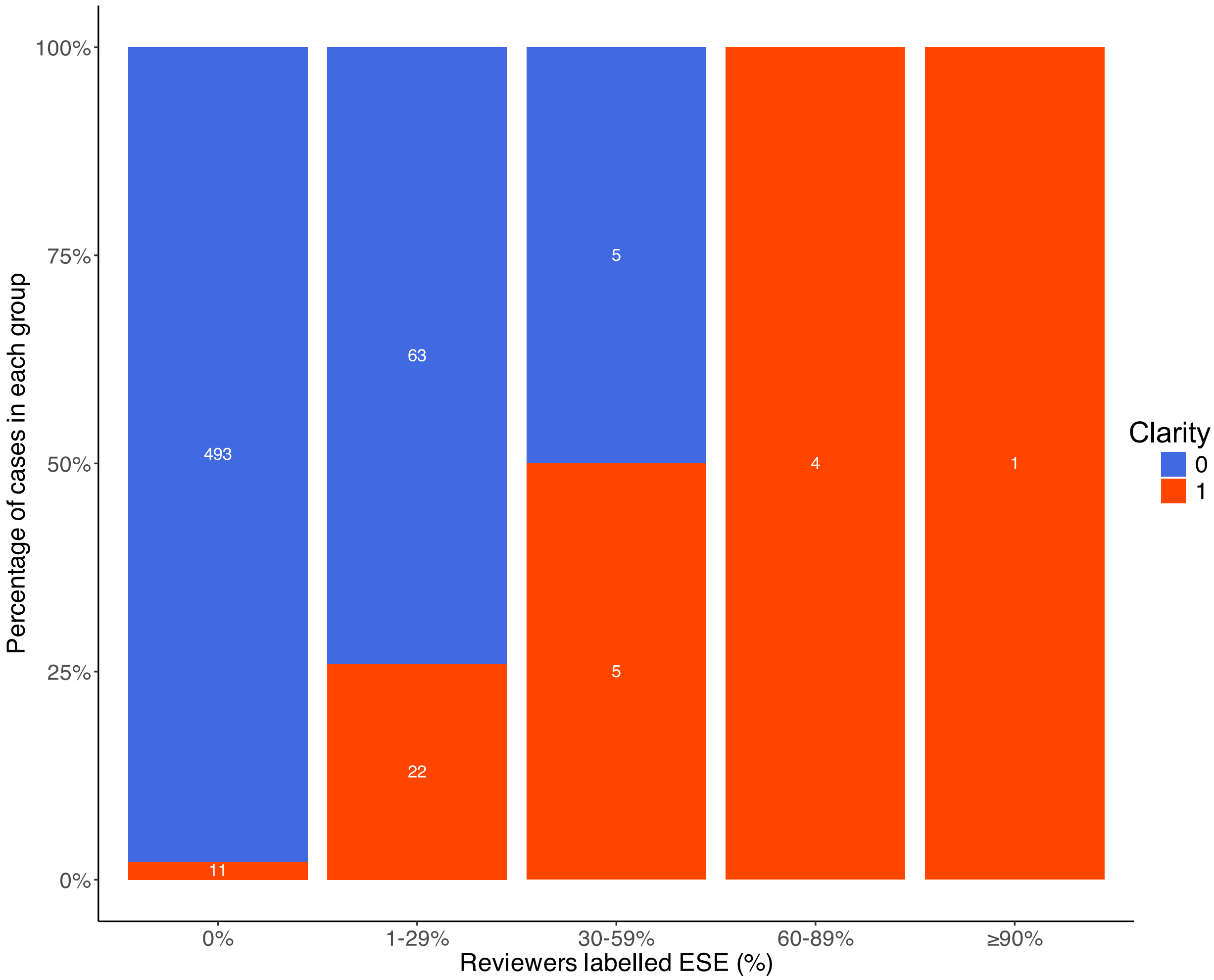

Supplementary Figure 1. Distribution of cases grouped by the percentage of reviewers that agreed ESE was present, and their corresponding AI algorithm (Clarity) output. Label of 0 = no ESE, 1 = ESE present by the algorithm

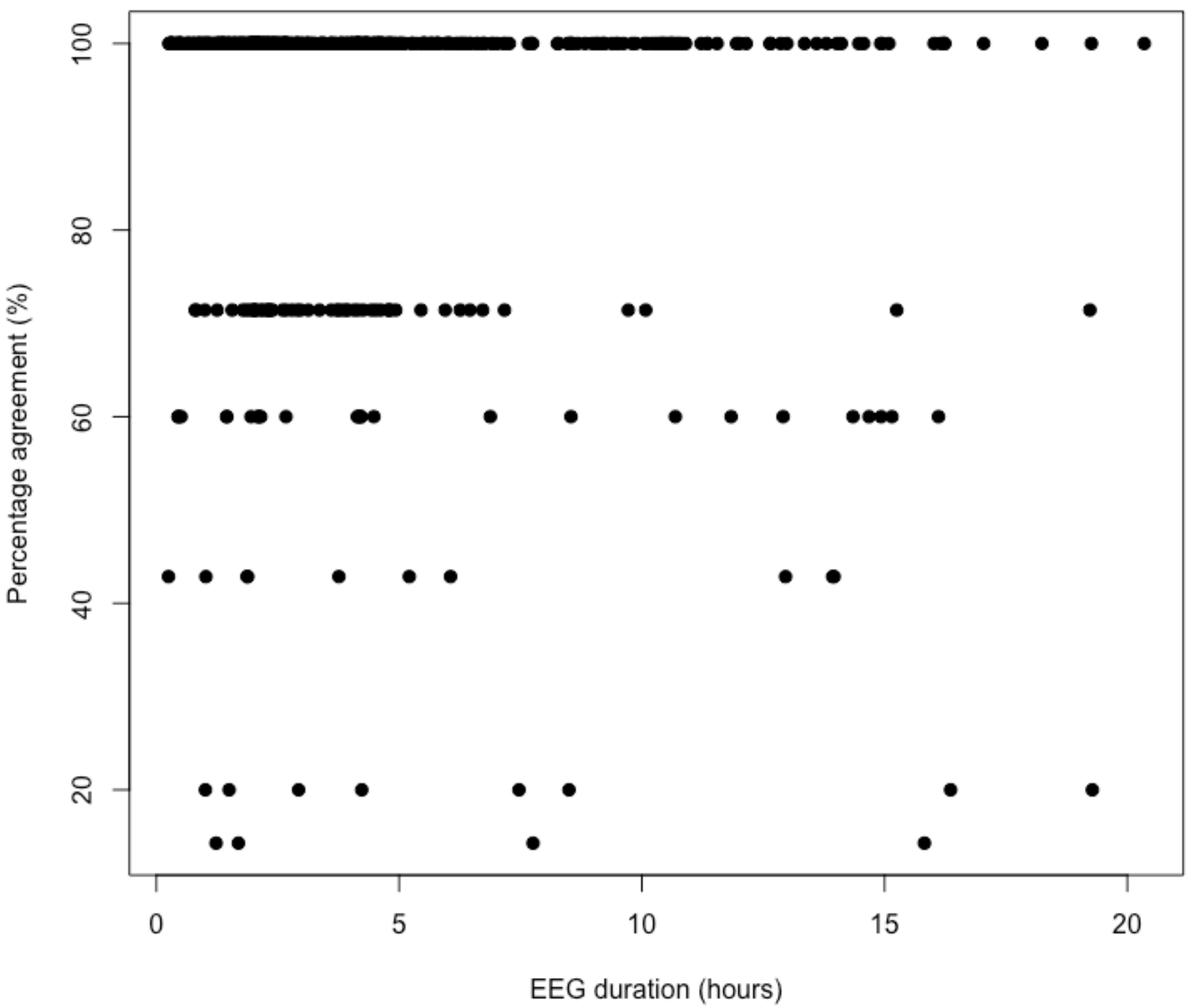

Supplementary Figure 2. Distribution of EEG duration (horizontal axis) versus the percentage of agreement (vertical axis) among all available reviewers. Reviewers were considered to be in agreement if they determination of ESE/no-ESE matched.

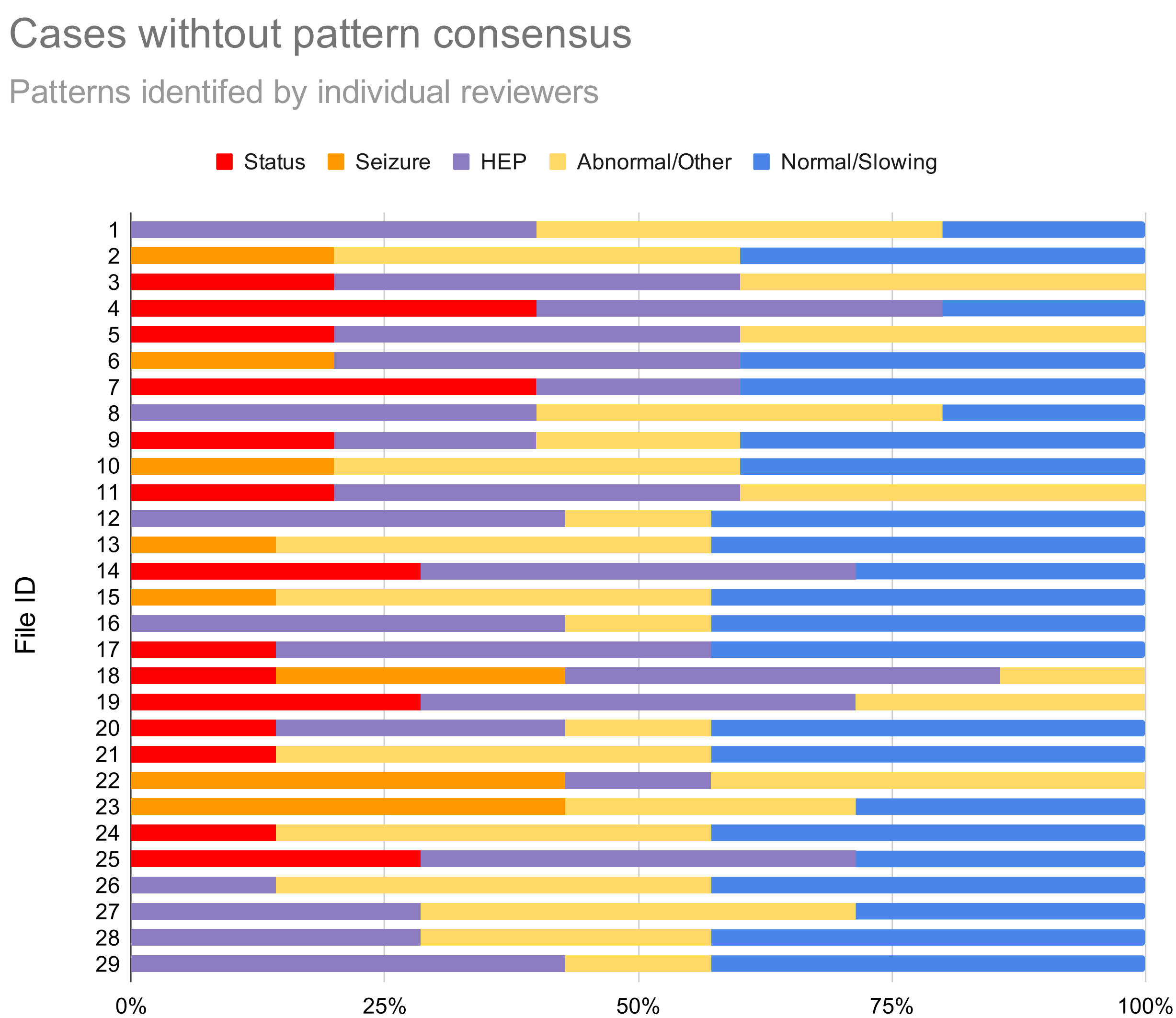

Supplementary Figure 3. Breakdown of patterns identify by reviewers in cases were no majority consensus was reached.

	label		
Predictors	*Odds Ratios*	*CI*	*p*
(Intercept)	0.00	0.00 – 0.00	**<0.001**
duration	1.09	0.93 – 1.28	0.277
experience1-10	0.22	0.02 – 2.38	0.212
location [ICU/Floor]	0.94	0.21 – 4.12	0.932
**Random Effects**			
σ^2	3.29		
$\tau_{00\ \mathrm{fileID}}$	136.45		
$\tau_{00\ \mathrm{revID}}$	2.22		
ICC	0.98		
N_{fileID}	602		
N_{revID}	7		
Observations	3774		
Marginal R^2 / Conditional R^2	0.005 / 0.977		

Supplementary Figure 4. Summary of Generalized Mixed Effects Model in ESE label determination. The fixed effects included the duration of recording in hours ("duration"), whether the reviewers had 1-10 years of experience or more ("experience1-10"), and where the EEG was acquired ICU/Floor vs. ED ("location[ICU/Floor]"). The random effects were the EEG file ("fileID") and the individual reviewers ("revID") .CI: Confidence Interval; ICU: Intensive Care Unit.

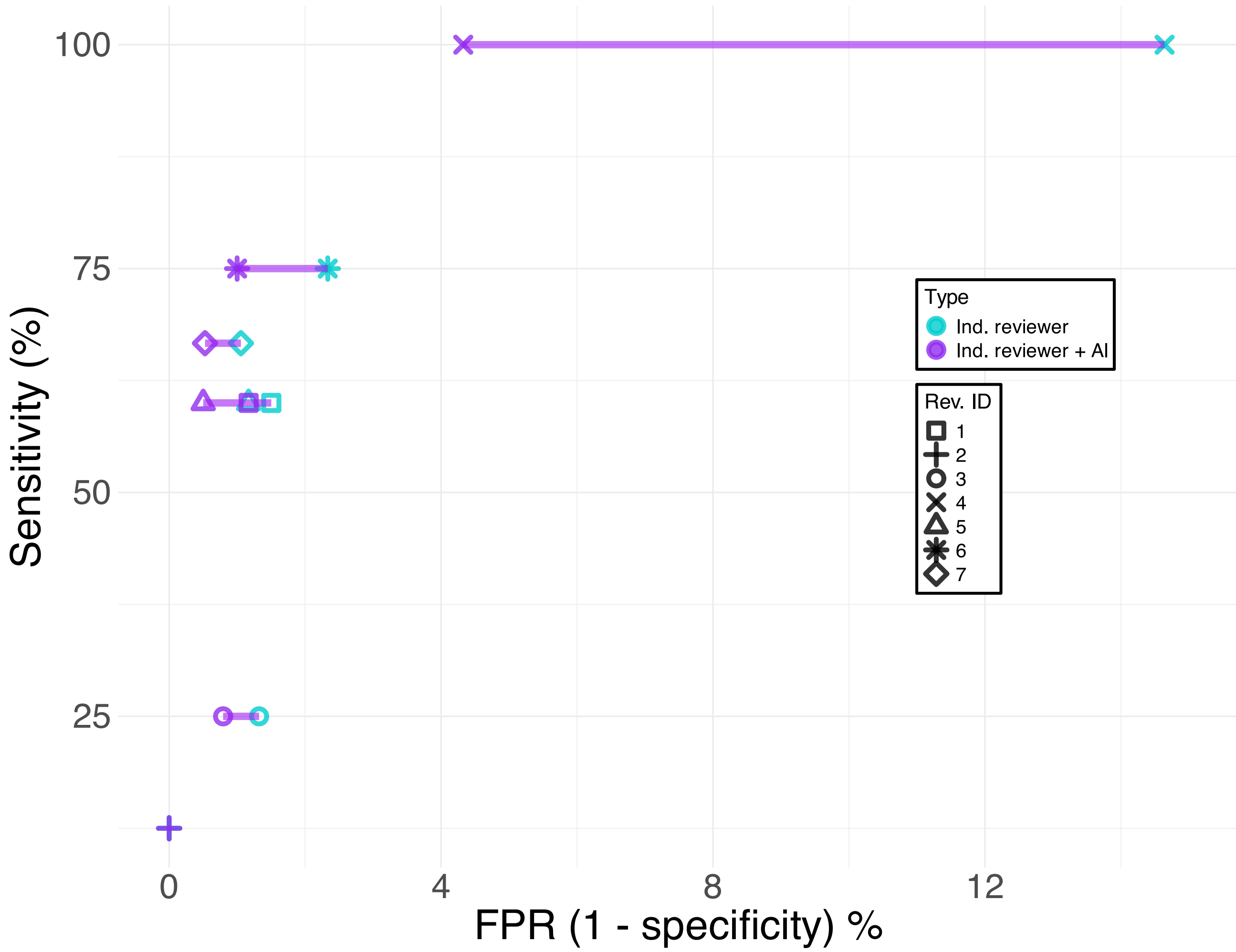

Supplementary Figure 5. Comparison of individual reviewers' performance for detection of ESE in leave-one-out analysis from the original response (cyan markers) vs. simulating AI+Human collaboration (purple markers). Each marker symbol represents the reviewer who was "left out" on the group who generated the majority consensus.

References

1. Hirsch LJ, Fong MWK, Leitinger M, LaRoche SM, Beniczky S, Abend NS, et al. American Clinical Neurophysiology Society's Standardized Critical Care EEG Terminology: 2021 Version. Journal of Clinical Neurophysiology. 2021;38(1).
